# Overcoming stalled translation in human mitochondria

**DOI:** 10.3389/fmicb.2014.00374

**Published:** 2014-07-18

**Authors:** Maria T. Wesolowska, Ricarda Richter-Dennerlein, Robert N. Lightowlers, Zofia M. A. Chrzanowska-Lightowlers

**Affiliations:** Wellcome Trust Centre for Mitochondrial Research, Institute for Cell and Molecular Biosciences, Newcastle University, Medical SchoolNewcastle upon Tyne, UK

**Keywords:** mitochondria, release factor, ICT1, ribosome rescue, ribosome stalling, protein synthesis, translation

## Abstract

Protein synthesis is central to life and maintaining a highly accurate and efficient mechanism is essential. What happens when a translating ribosome stalls on a messenger RNA? Many highly intricate processes have been documented in the cytosol of numerous species, but how does organellar protein synthesis resolve this stalling issue? Mammalian mitochondria synthesize just thirteen highly hydrophobic polypeptides. These proteins are all integral components of the machinery that couples oxidative phosphorylation. Consequently, it is essential that stalled mitochondrial ribosomes can be efficiently recycled. To date, there is no evidence to support any particular molecular mechanism to resolve this problem. However, here we discuss the observation that there are four predicted members of the mitochondrial translation release factor family and that only one member, mtRF1a, is necessary to terminate the translation of all thirteen open reading frames in the mitochondrion. Could the other members be involved in the process of recycling stalled mitochondrial ribosomes?

## INTRODUCTION

Maintaining the efficiency and accuracy of protein synthesis is one of the most important aspects of cell survival. The translation of mRNAs into polypeptides is a complex multistep process that involves many proteins and RNA species. Consequently there are many points at which protein synthesis can be disrupted with consequent detrimental effects on cell viability (Zaher and Green, 2009). One step at which this process can fail is when the ribosome ceases to progress along the open reading frame within the transcript, termed stalling. The reasons for this are multiple and varied. Elongation arrest can be an important regulatory step such as is seen in the binding of signal recognition particles (SRP) to emergent nascent peptides. Docking of the SRP to its receptor in the endoplasmic reticulum (ER) membrane facilitates co-translational translocation and the nascent peptide is immediately inserted into the ER membrane prior to any folding event (Walter et al., 1981). Structural or sequence elements within the mRNA may cause pausing, as will the lack of sufficient charged tRNAs. In bacteria, examples of each of the events have been shown to trigger degradation of the mRNA on which the ribosome has paused (reviewed in Deana, 2005). In certain cases, however, there is potential for translational arrest to be more harmful, or more specifically that not alleviating the arrest or the cause of it, can be detrimental. For example, stalled ribosomes sequester tRNAs within the A, P, and E-sites thereby limiting their availability, impeding normal translation (Manley, 1978; Jørgensen and Kurland, 1990). Bacterial ribosomes can also stall by colliding with the RNA polymerase ahead of them, which has itself stalled on its template. The phenomenon of translational arrest caused by ribosome stalling appears to occur in bacteria and in the cytosol of eukaryotes, and although under-researched it is also likely to affect mitochondrial protein synthesis. Because of the detrimental effects that can result from stalling and the relative frequency of premature termination events, organisms have developed different strategies to rescue these ribosomes.

Eubacteria have developed a number of mechanisms (reviewed in Janssen and Hayes, 2012) but the best characterized is *trans*-translation promoted by tmRNA (reviewed in Moore and Sauer, 2007). This system, present in all eubacteria utilizes a molecule that folds to present two very different domains. The 5′ domain resembles a tRNA, which is recognized and aminoacylated by alanyl-tRNA synthetase. Aborted nascent peptides are transferred from the P-site tRNA to the alanine on this upstream tRNA-like structure, resulting in an (peptidyl)-alanyl-tmRNA. The downstream element of the tmRNA then acts as an mRNA, where the first triplet, or resume codon, generally encodes an alanine (Kapoor et al., 2011). Protein synthesis is resumed with the addition of an approximately 10 amino acid tag, before terminating in a conventional stop codon. The aberrantly translated peptide is able to leave the ribosome through the conventional mechanism, thereby rescuing the stalled components. This system is found in all known bacterial genomes, either a single tmRNA or as two pieces that bind to resemble tmRNA (Keiler et al., 2000). The elegance of this arrangement is that it relieves the stall and tags the truncated/aberrant protein, effectively targeting it as a substrate for degradation. *Trans*-translation also requires the essential binding partner SmpB, which with tmRNA rescues ribosomes stalled on RNA templates that either lack a stop codon or have stalled during the elongation phase for other reasons. Alternative rescue pathways identified in *Escherichia coli* require the activity of protein factors ArfA or ArfB (YaeJ) that both utilize translation termination mechanisms to release arrested ribosomes (Chadani et al., 2010; Handa et al., 2010b; Abo and Chadani, 2014). The two mechanisms differ. ArfA requires recruitment of the translation termination factor RF2, whilst YaeJ retains the GGQ motif characteristic of release factors (RFs) and directly stimulates the ribosome dependent catalysis of the ester bond between the peptide and the P-site tRNA (Chadani et al., 2012). In eukaryotes, no homologs of the tmRNA system have been found, but a different mechanism has been identified to tackle the same problem. Characterized in the yeast cytosolic compartment, this system employs the protein Dom34, a homolog of the eukaryotic release factor (eRF1) but lacking the characteristic GGQ motif and codon recognition capability (Lee et al., 2007; Graille et al., 2008). Dom34 can act in concert with either a GTPase, Hbs1, or an ATPase, Rli1. In a codon-independent manner, it releases ribosomes from truncated transcripts or those that have failed to release the mRNA at the stop codon and have migrated into the 3′UTR (Guydosh and Green, 2014). Although mitoribosomes are likely to encounter similar issues that cause stalls, similar to the eukaryote cytosol no tmRNA species has been found in mammalian mitochondria. Curiously, a circularly permuted gene resembling the upstream tRNA-like fragment was identified in the primitive mitochondrial genome of *Reclinomonas americana* (Keiler et al., 2000). However, since no accompanying open reading frame for the tag peptide could be found, it seems unlikely that any mitochondrial genome has retained this apparatus. We are left, therefore, with no mechanistic data on precisely how stalled ribosomal complexes are resolved in mitochondria.

## MITOCHONDRIAL RIBOSOMES, STALLING, AND PREDICTING POTENTIAL RESCUE MECHANISMS

Our understanding of all the critical recognition elements and *trans*-acting proteins responsible for mitochondrial translation lags behind the characterization in bacteria and the eukaryotic cytosol. Many of the aspects that are still unknown include the mechanisms that exert quality control of protein synthesis and rescue ribosome stalling. Given the presumed α-proteobacterial origin (Gray et al., 1999) of the organelle, the prediction is often that processes in mitochondria will strongly resemble those from their bacteria origins (Smits et al., 2010). The existing models of translation in mitochondria are, therefore, based on those of bacteria. However, despite evident similarities between the two processes, they are not identical and certain unique features of mitochondrial translation make direct comparison more complicated (reviewed in Christian and Spremulli, 2012).With respect to the ribosome rescue mechanisms, one important consideration is the structure and composition of the mitoribosome. Mitochondrial ribosomes are often compared to their prokaryotic counterparts (Sharma et al., 2003), however, mitoribosomes vary enormously depending on their organism of origin (Rackham and Filipovska, 2014). Although all consist of a small and a large subunit there can be variations in their size, RNA to protein ratio, and composition. Throughout their evolution, mitoribosomes have acquired many distinct structural characteristics, including the unusually high protein to rRNA ratio, caused by shortening of rRNA and recruitment of additional proteins (Sharma et al., 2003). Although many of the mitoribosome proteins (MRPs) have bacterial homologs, almost half of them are specific to mitochondria (Sharma et al., 2003; Koc et al., 2010). These unique MRPs are mostly situated on the outer surface of the mitoribosome some of which compensate for the loss of rRNA domains or missing bacterial proteins (Sharma et al., 2003). These new protein also form an extended peptide exit tunnel, the central protuberance and line the mRNA entry site, which differs in structure from the prokaryotic counterparts (Sharma et al., 2003; Greber et al., 2013; Kaushal et al., 2014). Recent publications describing high resolution cryo-electron microscopy (cryo-EM) structures of both the mammalian 39S large (Greber et al., 2013) and 28S small mitoribosomal subunits (Kaushal et al., 2014) confirm the unique aspects of mitoribosome architecture derived from these mitospecific RPs. Any potential ribosome rescue mechanism in mitochondria might be predicted to reflect these global changes to the structural features and composition of the mammalian 55S particle. However, analysis of mitochondrial proteins that have bacterial homologs with known function, and comparable structures may still be the best way to begin the search for potential mitoribosome rescue factors. Other approaches include looking for factors that transiently interact with the mitoribosome, or through bioinformatics analyses. Use of the last two methods have helped to identify the most likely candidates, namely members of the mitochondrial RF family (Rorbach et al., 2008; Richter et al., 2010).

## MITOCHONDRIAL RELEASE FACTOR FAMILY

There are two types of release factors: those that are capable of mRNA sequence recognition (class 1 RFs) and those that are not (class 2). Class 1 RFs effect translation termination by sampling the ribosomal A-site and remaining transiently associated when they recognize a cognate STOP codon. Their function is to release the completed polypeptide from the ribosome by catalyzing the cleavage of the ester bond between the P-site tRNA and the terminal amino acid of the nascent peptide. Eubacteria utilize two different RFs, RF1 and RF2, to recognize the 3 universal STOP triplets (Oparina, 2005). In contrast, archaebacteria and eukaryotic cytosol both contain a single, omnipotent class 1 RF (named aRF1 and eRF1 respectively) that recognizes all three of the canonical STOP codons, UAA, UAG, and UGA (Ito et al., 2002; Seit-Nebi et al., 2002). Large scale phylogenetic analysis has examined the evolution and diversification of RF proteins and identified that members are present in both plastids and mitochondria (Duarte et al., 2012).

Human mitochondria use only UAA and UAG as terminating triplets, as UGA has been recoded to tryptophan. In combination with the altered characteristics of the mammalian mitoribosome this might predict the need for a reduced number of RFs. It is perhaps a surprise, therefore, that bioinformatics classifies four proteins as members of the human mitochondrial RF family, namely mtRF1, mtRF1a, ICT1, and C12orf65. The first to be identified solely by database searches was mtRF1 (Zhang and Spremulli, 1998). The sequence recognition domains differed from the consensus, supporting the assumption that mtRF1 functioned as a single RF that recognized the four codons that at the time were assumed to function as stop codons. This premise was absorbed into the literature until mtRF1a was identified, with decoding domains that more closely resembled the consensus, and biochemical characterization confirming its recognition of UAA and UAG as stop codons (Soleimanpour-Lichaei et al., 2007). The second confounding assumption that had been accepted in the literature was that AGA and AGG were also stop codons. Since these followed the final coding triplet in mitochondrial transcripts *MTCO1* and *MTND6* respectively, this was not an unreasonable interpretation of the human mitochondrial genome (Anderson et al., 1981). More recent investigations in whole cells have shown that physiologically neither of these are stop codons. Although both codons are unassigned, they function to promote a -1 frameshift, to position UAG in the A-site for conventional termination by mtRF1a (Temperley et al., 2010).

Since mtRF1a is sufficient to terminate translation of all 13 open reading frames, what are the functions of the remaining 3 mitochondrial RF family members? Is there any evidence that they can still function as RFs? These proteins were grouped together due to similarities in their sequence and structures that they share with RFs from bacteria and the eukaryotic cytosol (Duarte et al., 2012). In particular all four family members display high conservation of the GGQ domain that is critical for catalyzing peptidyl-tRNA hydrolysis (PTH; Frolova et al., 1999). For the RF to trigger ester bond cleavage the GGQ domain must be positioned in the peptidyl transferase center of the ribosome (PTC), which occurs when the RF undergoes a major conformational change from a closed to open conformation (Vestergaard et al., 2001; Petry et al., 2005; Laurberg et al., 2008). In order to prevent RFs from displaying PTH activity too early, the conformational change that promotes peptidyl-tRNA hydrolysis is dependent on stop codon recognition (Shaw and Green, 2007; Laurberg et al., 2008). The required sequence specificity is dictated by another conserved domain, which comprises amino acid stretches that come together in space. This domain deviates from the consensus in mtRF1, both in amino acid content and by being extended in length. The hypothesis predicated on these changes, is that the extra bulk of the sequence recognition domain can fill the space in the A-site normally occupied by the mRNA. Three dimensional modeling shows that mtRF1 could occupy this cavity and synchronously extend the GGQ motif into the PTC to rescue ribosomes that have stalled with an incomplete peptide anchored to a mis-processed or partially degraded mRNA lacking a termination codon (Huynen et al., 2012). In contrast the codon recognition domain is absent in both ICT1 and C12orf65, the two remaining members of the mitochondrial RF family. Despite this, the retention of the GGQ motif in all family members strongly suggests they have all retained the ribosome dependent ability to release peptides from a P-site anchored tRNA. That they play an important role in translation is further substantiated as intra-organellar protein synthesis is impaired when ICT1 or C12orf65 are depleted or mutated (Antonicka et al., 2010; Richter et al., 2010). These observations suggest that mtRF1, C12orf65, and ICT1 are likely to function on stalled ribosomes or large subunits with peptidyl-tRNA still anchored within, allowing them to be recycled for a new round of translation.

ICT1 is intriguing as it has been incorporated into the mitoribosome as a permanent fixture. This would appear to be dangerous, as ICT1 displays codon independent PTH activity, which *a priori* could cause premature peptide release (Richter et al., 2010). Since this does not occur physiologically, the associated PTH activity of ICT1 must be carefully controlled with an as yet undefined specificity. The current hypothesis is that ICT1 must function in ribosome rescue but whether this occurs at stalling events within ORFs or on truncated transcripts is not yet clear. ICT1 does, however, have a bacterial homolog, YaeJ, which has been shown to be involved in release of arrested ribosomes (Gagnon et al., 2012). Below we will compare and contrast and see if there are useful parallels to be drawn to elucidate the potential role of ICT1 in mitochondrial ribosome rescue.

## YaeJ

ArfB or YaeJ, is conserved among eukaryotes and is present in many Gram-negative species (Handa et al., 2010b). Its potential role in ribosome rescue was indicated by its structural similarity to RF1 and RF2 and the presence of the GGQ motif, characteristic of ribosome dependent PTH activity (Frolova et al., 1999). Convincing evidence for its function in ribosome rescue derived from studies in *E. coli* where YaeJ overexpression in strains lacking tmRNA and ArfA suppressed the lethal phenotype (Chadani et al., 2011). Subsequently YaeJ was shown to have direct PTH activity on stalled ribosomes both *in vitro* (Handa et al., 2010b) and *in vivo* (Handa et al., 2010b; Chadani et al., 2011), which was lost when the GGQ was mutated to GAQ (Chadani et al., 2011). This indicated that no auxiliary factors were required, in contrast to ArfA that needs to co-opt RF2 for activity. As with ICT1, the protein lacks domains 2 and 4 of a standard RF, thereby losing codon-recognition consistent with its ability to rescue ribosomes stalled on mRNA lacking STOP codons (Chadani et al., 2011). Structural analysis by the Steitz group has detailed the critical interactions that drive ribosomal rescue. The N-terminal globular domain is bound in the A-site and is joined to the C-terminus via a flexible linker (Gagnon et al., 2012). Although the C-terminus was thought to be an unstructured, it has a basic residue-rich tail that was necessary to facilitate interaction with the ribosome (Handa et al., 2010b; Chadani et al., 2011; Gagnon et al., 2012; Kogure et al., 2014). Gagnon et al. (2012) have shown that once positioned within the mRNA entry channel, it forms an α-helix. Their data suggest that the YaeJ tail can sample the mRNA channel and thereby determine whether or not the ribosome has stalled on a non-stop transcript or is still translating (Gagnon et al., 2012). If the ribosome is stalled on non-stop mRNA or an endonucleolytically cleaved transcript, the basic residues of the YaeJ tail could interact with negatively charged rRNA nucleotides lining the tunnel. Such binding to the ribosome would cause structural rearrangements within YaeJ, similar to those following codon recognition of a standard RF, placing the GGQ domain within the PTC, facilitating peptidyl-tRNA hydrolysis (Gagnon et al., 2012). The data is clear that YaeJ is an important protein in ribosome rescue, but does it follow that the human mitochondrial ortholog plays a similar role?

## ICT1

First reported, under the name of DS-1, as a transcript downregulated during *in vitro* differentiation of a colon carcinoma cell line, immature colon carcinoma transcript-1 (ICT1; Van Belzen et al., 1995, 1998) had no connection to any potential mitochondrial function. Subsequent research by our group investigating ribosome recycling in human mitochondria, identified ICT1 as associated with mtRRF (Rorbach et al., 2008). Subsequently, the available data and bioinformatic analyses classified ICT1 in the prokaryote/mitochondrial RF family (uniprot Q14197). At ∼175 amino acids post-maturation ICT1 is smaller than standard RFs due mainly to the loss of the codon recognition elements. It does, however, retain the GGQ motif that has been confirmed as functional, as GSQ and AGQ mutations affect growth and activity (Richter et al., 2010). As mentioned above, this work confirmed that ICT1 is not just mitochondrial but has actually become an integrated component of the large ribosomal subunit. Recent cryo-EM coupled with cross-linked mass spectroscopy confirmed this observation and positions ICT1 at the central protruberance, close to MRPL15, -18, and -49 (Greber et al., 2013). Perhaps not surprisingly, depletion of ICT1 causes disruption of the mitoribosomal structure and subsequent *de novo* synthesis of mitochondrially encoded proteins (Richter et al., 2010). The combination of these characteristics make ICT1 a codon-independent but ribosome-dependent and ribosome-integrated peptidyl-tRNA hydrolase.

Analysis of ICT1 solution structure may provide more insight into the role of the protein (Handa et al., 2010a). The three distinct regions are the N-terminal mitochondrial targeting signal, the structured catalytic domain containing the GGQ motif, and an unstructured C-terminal extension rich in positively charged amino acids. The topology of the GGQ domain is β1-β2-α1-β3-α2, which follows the bacterial RF2 pattern with the exception of the α1 inserted between β2 and β3 that is not present in the latter (Handa et al., 2010a). Loss of the codon recognition domain appears to be replaced by a C-terminal extension, somewhat reminiscent of YaeJ.

## YaeJ vs. ICT1

**FIGURE 1:**
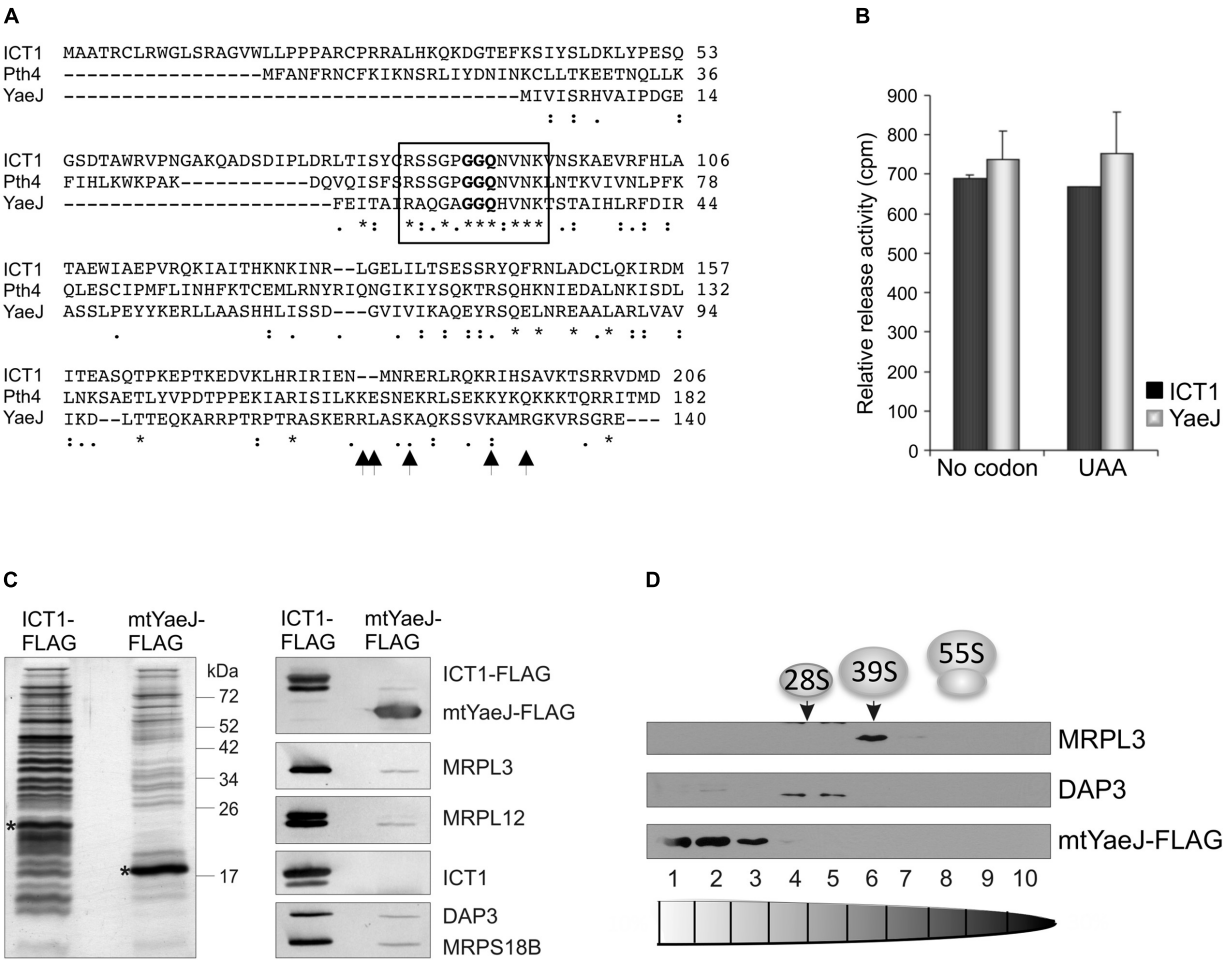
**ICT1 as a mitochondrial translation rescue factor and its possible orthologs. (A)** *Alignment of human ICT1 (14197) with the Pth4 ortholog from S. pombe (Q9HDZ3) and YaeJ ortholog from E. coli (E2QFB9).* Identity is indicated by (*), high levels of similarity by (:) and lower levels by (⋅). The conserved GGQ region is boxed and the YaeJ residues that are required for PTH activity and are highly conserved between bacterial species are indicated by arrows. **(B)** *Release activity of recombinant YaeJ and ICT1.* PTH activity was tested on 70S ribosomes primed with either no RNA or UAA triplet in the A-site. **(C)** *Mitochondrial targetted YaeJ-FLAG shows interaction with human mitochondrial ribosomal proteins*. FLAG tag mediated immunoprecipitations of ICT1 and mitochondrially targeted YaeJ were performed on lysates of HEK293 cell lines induced for 3 days. The elution fractions (10%) were separated by SDS PAGE and analyzed by silver staining (left panel, FLAG protein indicated by *) or western blot (right panel). Antibodies against MRPL3, MRPL12, ICT1, DAP3, and MRPS18B were used to determine the relative levels of coimmunoprecipitated ribosomal proteins. The presence of FLAG tagged protein in each elution was confirmed by anti-FLAG antibodies. **(D)** *Mitochondrially-targeted YaeJ-FLAG does not co-migrate with the 39S LSU.* Lysate (700 μg) of mtYaeJ-FLAG expressing cells was separated on an isokinetic sucrose gradient. Fractions were analyzed by western blot using antibodies against the 39S LSU (MRPL3) and the 28S SSU (DAP3). The distribution of mtYaeJ-FLAG was determined by applying FLAG antibodies. Methods for panels **(B–D)** were essentially as described in Richter et al. (2010), except a YaeJ-FLAG construct was used to generate a HEK293T overexpression line instead of the ICT1 FLAG.

**FIGURE 2:**
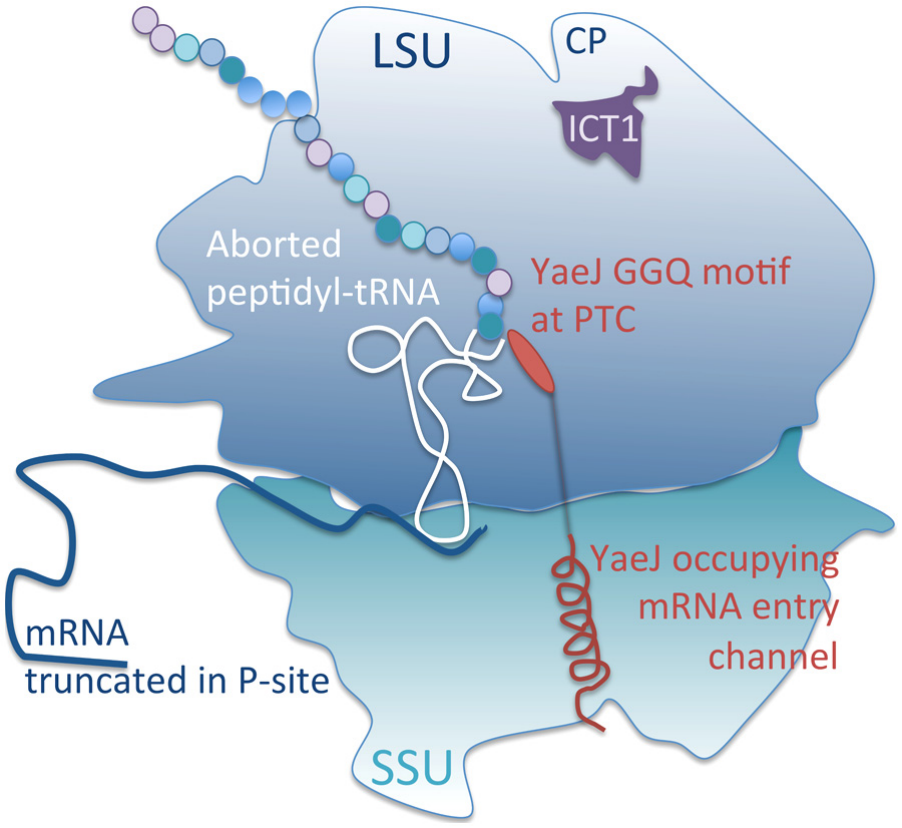
**Schematic of a composite ribosome to show relative positions occupied by ICT1 and YaeJ.** Truncated mRNA lacking an A-site codon allows ingress of YaeJ such that the C-terminal α-helix aligns within the mRNA entry channel in the small subunit (SSU). This positions the GGQ motif at the peptidyl transferase centre (PTC) allowing cleavage of the ester bond between the P-site tRNA and the truncated polypeptide (as described in Kogure et al., 2014). The aborted product is then released via the polypeptide exit site in the large subunit (LSU). ICT1 by contrast is located at the central protuberance (CP) precluding its interaction with the nascent peptide without a large scale conformational change of the ribosome (as described in Greber et al., 2013).

Comparisons of ICT1 and YaeJ structure and sequence alignment show both similarities and differences (**Figure 1A**; Handa et al., 2010a; Gagnon et al., 2012; Kogure et al., 2014). Identity and similarity are strong in the GGQ domains. The N-termini differ as would be expected, as ICT1 is a mitochondrially destined protein. Although an inserted region (α_i_) is common to both the structure that follows differs, in YaeJ this is a β-strand in contrast to a 3_10_ helix in ICT1 (Kogure et al., 2014). The inserted α-helices share structure but differ in amino acid sequence, they are a characteristic and conserved feature for these two proteins that distinguish them from class I RFs (Kogure et al., 2014). An important feature for ribosome binding and activity in YaeJ was the length and amino acid composition of the linker and C-terminal region and critical residues for PTH activity are conserved between ICT1 and YaeJ (Kogure et al., 2014). The conserved similarities that link these two proteins whilst simultaneously distinguishing them from other RFs, suggest a conserved function and mechanism. As with Kogure et al. (2014) we could show codon-independent release activity by using recombinant YaeJ in *in vitro* assays with 70S ribosomes (**Figure 1B**). We, therefore, looked to see whether the similarities in these proteins were sufficient for YaeJ to substitute for ICT1 in the mitoribosome. To test this hypothesis, we generated cell lines that could inducibly express a mitochondrially targeted YaeJ (reported here) or the potential yeast mitochondrial RF, *Schizosaccharomyces pombe* Pth4 (reported in Dujeancourt et al., 2013). Each was generated with a C-terminal FLAG tag to facilitate efficient immunoprecipitation (IP). The immunoprecipitated protein was specifically and competitively eluted using FLAG peptide and in ach case demonstrated an association with the mitoribosome, but neither was as efficient as ICT1 at immunoprecipitating MRPs (**Figure 1C**). Each cell line was induced to express either YaeJ or Pth4 and the cell lysates were separated by isokinetic sucrose gradient (as in Richter et al., 2010). In neither case did the expressed protein migrate in fractions with the mt-LSU polypeptides (**Figure 1D**). Similar results were derived for mtRRF-FLAG (Rorbach et al., 2008), supportive of an interaction with mitoribosome mediated by transient A-site entry, akin to YaeJ interaction with the bacterial ribosome (**Figure 2**; Gagnon et al., 2012; Kogure et al., 2014) rather than mitoribosome integration. This suggests that these proteins may take part in ribosome rescue but by different mechanisms. The integration of ICT1 into the mitoribosome and the cryo-EM data positioning it near the central protuberance would preclude unrestricted access of the GGQ motif to the PTC (Greber et al., 2013). This might indicate the pathway in which ICT1 is involved, as without significant conformational changes in the mitoribosome it would not be able to exert PTH activity (**Figure 2**). Such structural rearrangements of the 55S might potentially occur to release tRNA from prematurely discharged peptidyl-tRNA complexes, or if subunit dissociation occurs prior to release of the peptide. Further data is required to confirm the substrate and mechanism of ICT1 in the rescue of stalled translation in human mitochondria.

## DISCUSSION

This “perspective” aims to highlight how perplexing post-transcriptional gene expression in mitochondria can be. Translation will not be an error free process but the exact nature of those errors, have yet to be determined. Mitochondrial ribosomes from different organisms can vary dramatically, and those in mammals are currently the most significantly different from the norm. This alone would be enough to stymie progress but the lack of a robust transfection mechanism and the lack of a robust *in vitro* translation system makes study of mammalian mitochondrial translation processes a technical quagmire.

Lessons that we learnt from bacterial studies are unfortunately limited, as described above. Despite similarities between YaeJ and ICT1 and their common divergence from standard RFs, we cannot assume a similar mechanism using the bacterial paradigm, as the integration of ICT1 into the mitoribosome excludes a similar mechanism of action. Since bacteria have more than one rescue pathway, it seems probable that mitochondria will too. Thus far bioinformatics has narrowed the plausible candidates for mitochondrial rescue factors to members of mitochondrial RF family. In addition to ICT1, the feature conferring ribosome dependent PTH activity, the GGQ motif, is present in mtRF1 and C12orf65, neither of which have characterized functions. The latter shares sequence similarities with ICT1 (Kogure et al., 2012), moreover, evidence for its importance comes from clinical data where patients harboring mutations in C12orf65 manifest clear defects in mt-protein synthesis (Antonicka et al., 2010; Shimazaki et al., 2012; Spiegel et al., 2014). However, as discussed above, sharing similarities is not sufficient to infer function and confirmation of mtRF1 and C12orf65 as mitoribosome rescue factors will require evidence of their direct involvement in relieving ribosome stalling. This cannot be accomplished without initially developing a method to analyse stalled mammalian mitoribosomes. A promising approach to allow precisely such analyses comes from recent data using a patient cell line with a mutation in *MT-TY,* the gene encoding mitochondrial tRNA^Trp^. Greater accumulation of mitoribosomes on Trp codons was detected, inferring mitoribosome arrest due to the shortage of the aminoacylated wild type mt-tRNA^Trp^ (Rooijers et al., 2013). We are currently analysing mitoribosome distribution in the absence of the potential rescue factors, to confirm whether or not they indeed play a role in alleviating mitoribosome stalling.

## AUTHOR CONTRIBUTIONS

Maria T. Wesolowska – performing the mitoribosome profiling described in the discussion and assistance in writing the manuscript. Ricarda Richter-Dennerlein – performed the experimental work reported herein. Robert N. Lightowlers – contributed to the manuscript, design of experiments and grant holder of funding supporting the co-authors. Zofia M. A. Chrzanowska-Lightowlers – main contributor to the manuscript, design of experiments and grant holder of funding supporting the co-authors.

## Conflict of Interest Statement

The authors declare that the research was conducted in the absence of any commercial or financial relationships that could be construed as a potential conflict of interest.
